# Coffee Breeding and Stress Biology

**DOI:** 10.3390/plants13141912

**Published:** 2024-07-11

**Authors:** Fábio Luiz Partelli, Henrique Duarte Vieira

**Affiliations:** 1 Department of Agricultural and Biological Sciences, Centro Universitário Norte do Espírito Santo (CEUNES), Universidade Federal do Espírito Santo (UFES), Rodovia BR-101, Km 60, Litorâneo, São Mateus 29932-900, ES, Brazil; 2 Plant Technology Laboratory, Centro de Ciências e Tecnologias Agropecuárias (CCTA), Universidade Estadual do Norte Fluminense—Darcy Ribeiro (UENF), Avenida Alberto Lamego 2000, Bairro Parque Califórnia, Campos dos Goytacazes 28013-602, RJ, Brazil

## 1. Introduction

Currently, 130 species of the genus *Coffea* have been identified [1]. However, only two are cultivated on a large scale commercially. Of these, *C. arabica* L. (Arabica coffee) accounts for the majority of production (56%) and *C. canephora* L. Pierre ex A. Froehner (Robusta and Conilon coffees) account for the remaining 44% [2]. Over the last 30 years, global coffee production has significantly increased, peaking in 2019 at approximately 10.3 million tons. During this period, the contribution of *C. canephora* rose from about 30% in the 1990s to be 44% of total coffee production in 2022 [3].

In the past five years, domestic consumption—coffee consumed within the producing countries—amounted to around three million tons of dry beans, with the remaining amount mainly exported to Europe and North America, which together consumed almost 50% of all the coffee produced globally [2]. South America is leading in worldwide coffee production, primarily due to their high output of *C. arabica*. In the last five harvests, Brazil and Colombia have been among the top five global producers [3]. Brazil accounts for about 35% of the total production on the planet, combining both *C. arabica* and *C. canephora*.

In addition to differences in appearance and origin, these two species also vary significantly in the quality attributes of their beverages, namely, their body, aroma, and flavor [2,4,5]. Unlike *C. arabica*, which is self-pollinating, *C. canephora* is cross-pollinating and is cultivated by combining compatible genotypes [6]. *Coffea canephora* is one of the bases for the development of *C. arabica*, which originated from a natural hybridization event between the ancestors of the current *C. canephora* and *C. eugenioides*. This hybridization likely occurred between 10,000 and one million years ago [7,8].

As climate patterns evolve, technology advances, and consumer demands grow, the need for ongoing research into coffee becomes clear. In view of this scenario and aiming to broaden the discussion to include farmers, scientists, and the industry, this Special Issue makes a significant contribution to this effort. We are pleased to present nine articles in the thematic series “Coffee Breeding and Stress Biology”.

## 2. Published Articles

This Special Issue compiles a broad array of studies and research perspectives united by a common theme: coffee cultivation. It includes seven scientific articles and two review papers:

The first study to be reported is by Bezerra et al. [9]. This research was conducted in Manaus, AM, Brazil, using *C. canephora*. The authors quantified nutrient concentrations in various plant organs using total reflection X-ray fluorescence (TXRF). Genetic parameters and divergence were estimated, and genotypes were grouped using a hierarchical UPGMA method and non-metric multidimensional scaling analysis. The study differentiated between the performance of genotypes in absorbing essential and non-essential chemical elements for plant development and analyzed the correlation of these traits in the selection process. TXRF efficiently characterizes the presence and concentration of multiple elements, aiding in genotypic discrimination for the breeding of *C. canephora*.

The work by dos Santos et al. [10] aimed to explore the phenotypic plasticity of Timor Hybrid coffee germplasm. Conducted in a greenhouse, the experiment involved two conditions: one with normal soil moisture levels that were close to the field capacity, and another under a water-deficit scenario that included a period of drought followed by rehydration. The study reveals that the genotypes studied can significantly alter physiological and biochemical parameters in response to environmental stimuli and exhibit reduced biomass loss in shoots and roots when subjected to stress conditions.

Schmidt et al. [11] undertook an experiment in Alta Floresta D’Oeste, Rondônia, Brazil, involving Robusta coffee genotypes (*C. canephora*) grown in the Brazilian Amazon. Genetic variability was observed among the Robusta coffee genotypes, with VP06, AS4, and AS10 being the most dissimilar. LB080 had the lowest dry weight of fruits and the smallest percentage of grains relative to the husks. ZD156 accumulated more potassium in the grains, whereas VP06 and AS10 accumulated the most nutrients in the husks. The nutrients N, K, Ca, and P are accumulated in larger quantities, requiring the calibration of dosages and the distribution of mineral fertilizers.

Souza et al. [12] examined coffee plants grown in open chambers under high light (HL) or low light (LL) conditions (9 or 1 mol of photons m^−2^ day^−1^, respectively) and with ambient CO_2_ (aCa) or elevated CO_2_ (eCa) levels (437 or 705 μmol mol^−1^, respectively). The study demonstrated that most physiological traits were influenced by the light and the CO_2_ concentration, as well as by their interaction. In terms of aCa, (i) there was greater stomatal conductance (gs) (only under HL) with reduced diffusive limitations to photosynthesis; (ii) higher gs was noted during transitions from HL to LL, while gs did not respond to transitions from LL to HL regardless of [CO_2_]; (iii) there were increased leaf nitrogen pools (only under HL) and enhanced photosynthetic nitrogen use efficiency irrespective of light conditions; (iv) there was no evidence of photosynthetic acclimation; and (v) there was increased biomass allocation to roots and anterior branching. Thus, eCa improved plant growth and photosynthetic performance.

Soela et al. [13] sought to understand the interactions between spray quality and chlorophyll fluorescence variation when applying sunscreen to Conilon coffee plants. The study concluded that the nozzle and application rate did not directly affect the physiological responses of the plants. Plants without a sunscreen application exhibited high energy dissipation flux values. The performance index of photosystem II (PSII) and the maximum photochemical efficiency of the PSII indicated that using sunscreen on plants enhances photosynthetic activity and provides photoprotection against light stress, regardless of the application rate and spray nozzle used.

The study by Rodrigues et al. [14] aimed to examine the characteristics of coffee fruit (weight, percentage of husks/grains), determine the concentration and accumulation of nutrients in the fruits, grains, and husks of the coffee plant, and assess the genetic diversity among 20 genotypes of *C. canephora*. The study concluded that the nutrients most accumulated/exported in the fruits were N and K, respectively. There was significant genetic diversity among the 20 *C. canephora* genotypes studied regarding the nutrient concentration and percentage in grains and straw. Genotypes 8 and 1 were notable for having a higher proportion of grains, thus requiring the least amount of fruit to produce 1000 kg of ground coffee.

Covre et al. [15] employed a Bayesian approach to discriminate among 43 genotypes of *C. canephora* cv. Conilon grown in two producing regions, aiming to identify the most stable and productive genotypes. Conducted in southern Bahia and northern Espírito Santo, Brazil, the study revealed significant genetic divergence among the genotypes and detected significant effects due to the genotype, environment, and year. It was possible to discern differences between genotypes within the same environment. With a model yielding the lowest residuals, the study recommended the most productive genotypes for both environments: LB1, AD1, Peneirão, Z21, and P2.

The other two articles are reviews. The first, by de Oliveira et al. [16], describes recent advances in genome function and transcriptional control, highlighting small RNAs (sRNAs) that respond to environmental signals and can introduce variability through gene expression regulation. It discusses the predicted impact of climate change on coffee plants and the coffee production chain, emphasizing the role of sRNAs in response to environmental changes, particularly temperature, across different species, and their potential as tools for genetic improvement.

The second review, by Breitler et al. [17], explores agroforestry as a strategy to mitigate and adapt coffee cultivation to climate change. It describes that despite their benefits, agroforestry systems (AFSs) for coffee are not currently competitive, partly because all modern varieties are selected for intensive cultivation in full sun and exhibit low yields in shaded environments. It also discusses the potential coffee ideotypes required for AFS coffee cultivation, with the four main ones detailed in the review.

## 3. Conclusions

In total, the research presented in the seven articles and two review papers in this Special Issue highlights the essential role of ongoing research in the improvement of the species, plant nutrition, responses to climate change, and the management practices for coffee crops. These studies demonstrate potential for the improvement and sustainability of *Coffea canephora* and *C. arabica*, generating new scientific and practical knowledge.
